# A rare case of ascending colon actinomycosis mimicking cancer

**DOI:** 10.1186/1471-230X-5-1

**Published:** 2005-01-04

**Authors:** Dimitrios Filippou, Ioannis Psimitis, Diamanto Zizi, Spiros Rizos

**Affiliations:** 1 Department of Surgery, GP Hospital of Piraeus "Tzanion", Tzani & Afentouli str, Pireaus-Athens, Greece; 2 Department of Pathology, GP Hospital of Piraeus "Tzanion", Tzani & Afentouli str, Pireaus-Athens, Greece

## Abstract

**Background:**

Actinomycosis is a rare inflammatory disease caused by an anaerobic bacterium that can rarely affect the large intestine.

**Case presentation:**

We present a rare case of a cecum and ascending colon actinomycosis in a 72 years old woman, mimicking clinically a malignant inflammatory tumor of the right colon. The patient complained of right lower quadrant pain. Although our first thought was a peri-appendiceal abscess, CT scan suggested a right colon tumor. The patient underwent a right colectomy and the histological examination of the specimen revealed colon actinomycosis.

**Conclusions:**

Preoperative diagnosis in colon actinomycosis is difficult to achieve. Treatment of choice is antibiotics administration. A review of the possible pathogenesis and therapeutic modalities is also presented.

## Background

Actinomycosis is an uncommon inflammatory entity caused by the universally distributed anaerobic bacterium, Actinomyces Israeli. This Actinomyces is a gram-positive, rather microaerophilic bacterium, which consists a component of the normal human flora. Actinomyces requires the presence of many other bacteria, which destroy the over-vascularized regions and convert aerobic microenvironment to an anaerobic one. Then it's easy for Actinomyces to migrate, infect and proliferate in already injured tissue. Primary bowel involvement is rare, although it has been increased in frequency over the last years. The most common sites of the disease are the transverse colon and the cecum with the appendix [1,2].

Actinomycosis can mimic other abdominal diseases as diverticulitis, abscesses, inflammatory bowel disease and malignant tumors, presenting a diagnostic challenge, and identified post-operatively in most of the cases [3].

The treatment of choice is antibiotic administration, whenever it is possible due to diagnostic difficulties, although in most cases surgical intervention is performed Diagnosis can be achieved with endoscopy and imaging techniques, as computed tomography (CT scan) and magnetic resonance imaging (MRI). We present a rare case of right colon actinomycosis mimicking malignant tumor, causing bowel obstruction. The diagnosis was achieved only postoperatively.

## Case presentation

A 72 years old woman proceeded to the Emergency Department complaining of acute lower right abdominal pain, mild fever, mild weight loss and constipation. The past medical history of the patient was free.

**Figure 1:**
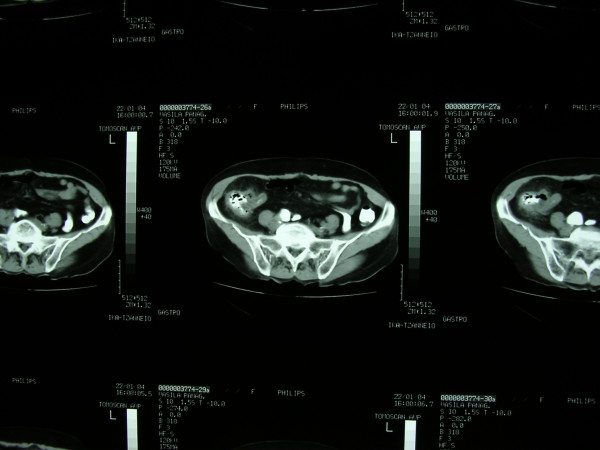
In abdominal CT scan a large inflammatory tumor like mass is identified. The characteristics of the mass do not indicate colon actinomycosis. The characters of the lesion and the coexisting edema and the severe and diffuse inflammation of the mesentery in the lower right quadrant probably suggest perforated ascending colon tumor.

The patient was presented with severe lower right abdominal pain, with signs of local peritonitis and a palpable mass in the same region. The body temperature was 37.2–37.4°C, while arterial blood pressure and cardiac rate 150/90 mmHg and 90/min respectively. The signs of local peritonitis combined with the palpable mass of the lower right abdominal area suggested perforation of the appendix and abscess formation. The laboratory examinations of the patient showed leucocytosis with white blood cell count (WBC) 19000 (with macrophage prevalence: 89%). Colonoscopy revealed obstruction of the right colon. Biopsies were not acquired because the colon lumen was obstructed and the endoscope could not approach the lesion. Computerized tomography (CT) scans of the abdomen (Figure 1) revealed a soft lobular mass, measuring 5 × 5 cm, attached to the ascending colon and cecum, compatible with a tumor. The most possible diagnosis was that of a perforated colonic tumor. The patient underwent explorative laparotomy and right hemicolectomy with an end-to-end ileocolonic anastomosis. Thorough exploration of the abdominal cavity revealed no other pathologic findings.

The surgical specimen consisted of a 10 cm length of the terminal ileum and the whole of the right colon. The serosa was very heavily covered by suppurative exudates and fibrotic tissue.

**Figure 2:**
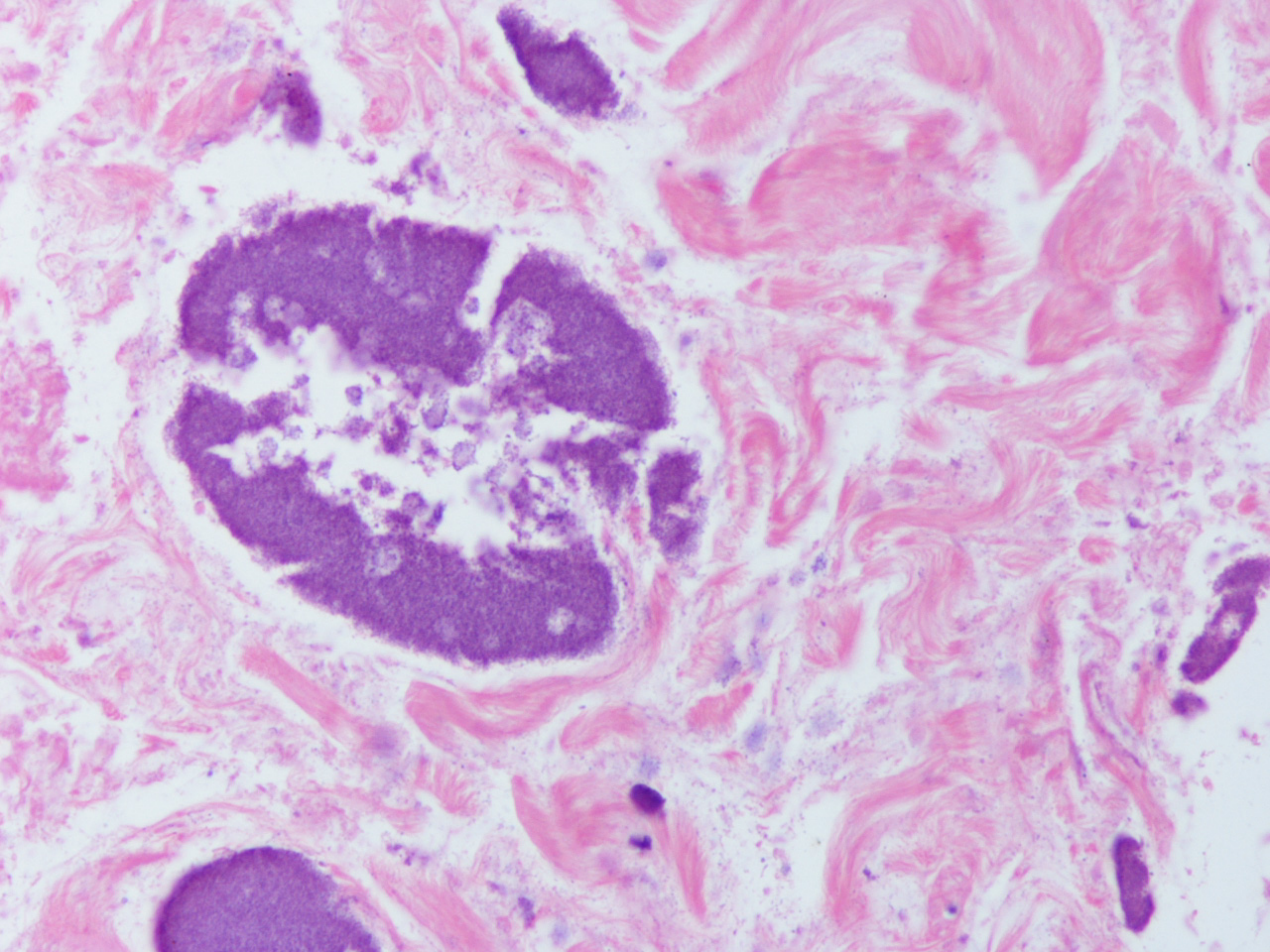
The histological examination of the removed specimen (right hemicolectomy specimen) revealed actinomyces colonies in the upper part of the cecum and the ascending colon wall. The large spherical clusters, densely packed and branching, and PAS positive "sulfur granules" are specific of actinomycosis.

Microscopic examination of the surgical specimen revealed thickening of the ascending colon wall with neutrophilic infiltration. Numerous polymorphonuclear leukocytes within the muscularis and a fibro-purulent reaction over the serosa with actinomycotic "sulfur granules" in it, were found in high power magnification (Figure 2).

Upon receiving the pathology report, systemic intravenous penicillin treatment was initiated. Therapy continued for 10 days and then followed by oral penicillin for 6 months. No postoperative complications were observed and the patient was discharged the 14^th ^day.

## Discussion

Actinomyces Israeli, a filamentous, gram-positive bacillus, is a constant part of the micro flora in the human oral cavity [4].

Actinomycosis presents a worldwide distribution and no sex predilection is obvious although most of the reported cases refer to males. Abdominal involvement occurs in only 20 percent of all cases of actinomycosis and can mimic malignancy, tuberculosis and inflammatory bowel disease [5].

Actinomyces is not always pathogenic, and normally exists in stagnated cecum or sigmoid colon. Predisposing factors include previous abdominal surgical operations, intestinal necrosis, foreign bodies, appendicitis and perforation. Some authors suggest that inflammatory or neoplastic processes may contribute to actinomycosis development [6,7].

Bowel obstruction and perforation due to actinomycosis without predisposing factors is very rare and only few cases have been described in the literature. Most commonly actinomycosis occurs in terminal ileus and appendix and rarely in the ascending colon, which is difficult to get obstructed. In our case actinomycosis affected cecum and ascending colon, with a dramatic clinical presentation. We searched the literature in Medline from 1997 to 2004 and we found that only a few cases with clinical presentation similar to our case have been reported.

Preoperative diagnosis is difficult although in some cases colonoscopy and histological examination of endoscopically acquired specimen can set the diagnosis. In our case the colon lumen was obstructed and no biopsies were taken. The CT findings suggested perforated colon tumor and an oncologic right hemicolectomy was performed. Actimomycosis usually mimics subacute infections or malignant tumors and the radiologic diagnosis of this entity may be difficult. Some authors suggest that abdominal CT scan with contrast enhancement may reveal a solid mass (intraluminal or extraluminal) with focal areas of attenuation invading the adjacent tissues and suggesting the diagnosis [8,9].

Most common findings in CT scan and/or barium study include mural invasion with stricture formation, mass effect with tapered narrowing of the lumen, and thickened mucosal folds. In many cases the radiologic findings are similar to those of Crohn's disease, intestinal tuberculosis, and excavated malignant tumors [10,11]. The most important CT feature for the correct diagnosis is a large mass adjacent to the involved bowel, which is also a very common finding in patients with colon actinomycosis. In rectosigmoid, colon cystic masses are more common, whereas in transverse or ascending colon purely solid masses are the predominant finding [12,13].

Goldwag et al suggest that CT guided fine needle aspiration can be both diagnostic and therapeutic. Microbiological analysis of material acquired by FNA may reveal sulfur granules, which are suggesting actinomycosis and nocardiosis. In most of the cases the sample receive is difficult especially when intestinal and colon are involved. We believe that in cases where the CT findings are non-specific, surgical exploration is necessary not only for diagnostic but also for therapeutic reasons [14].

High dose intravenous penicillin injection followed by orally administered penicillin for at least 6–12 months is the treatment of choice. Penicillin administration decreases morbidity and the patient may avoid an unnecessary operation [15,16].

Correct diagnosis is difficult and can be achieved preoperatively in only 10% of the cases, but it is of great importance because the appropriate treatment includes primarily penicillin administration. Surgical intervention is indicated only in cases with obscure diagnosis and for necrotic debridement removal. Although diagnosis only with imaging techniques and laboratory tests is difficult, abdominal actinomycosis should always be included in the differential diagnosis in patients with abdominal masses [1].

## Conclusions

In conclusion, colon actinomycosis should always be included in the differential diagnosis of abdominal masses with tumor of inflammatory characteristics. It should be especially suspected when the appearance on CT scan is of a solid mass with focal areas of attenuation or a cystic mass with a thickened wall showing inhomogeneous contrast enhancement that tends to invade adjacent tissues or structures. Immediate and accurate diagnosis, usually by FNA and cytology examination can prevent unnecessary surgical treatment.

## Competing interests

The author(s) declare that they have no competing interests.

## Authors' contributions

All authors contributed equally to this work. All authors read and approved the final manuscript.

## Pre-publication history

The pre-publication history for this paper can be accessed here:
